# Matching cues to attention: how visual and social cues differentially benefit students with low sustained or selective attention

**DOI:** 10.3389/fpsyg.2026.1848365

**Published:** 2026-07-23

**Authors:** Yao Li, Xuejiao Du

**Affiliations:** School of Education, Ludong University, Yantai, China

**Keywords:** instructional videos, selective attention, social cues, sustained attention, visual cues

## Abstract

**Background:**

In the digital era, fragmented information impairs the sustained and selective attention of some students, making it difficult for them to remain engaged in instructional videos. Adding cues to instructional videos is a direct method for attracting and sustaining attention. Cues can be classified as visual or social. However, the differential effects of visual and social cues on the learning outcomes of students with varying levels of sustained or selective attention remain unclear. To address this, two experiments were conducted with university students as participants.

**Methods:**

In both experiments, students watched instructional videos with different cues and completed test materials. Experiment 1 adopted a 2 (sustained attention level: high, low) × 2 (cue type: visual, social) between-participants design, with a final sample of 105 participants. Experiment 2 adopted a 2 (selective attention level: high, low) × 2 (cue type: visual, social) between-participants design, with a final sample of 104 participants.

**Results:**

The results of Experiment 1 showed that in the transfer test only, students with low sustained attention performed better with social than with visual cues. The results of Experiment 2 showed that in the retention test alone, students with low selective attention performed better with visual than with social cues.

**Conclusion:**

For students with low sustained attention, incorporating social cues into instructional videos may be a better choice than visual cues for promoting knowledge transfer. For students with low selective attention, incorporating visual cues may be prioritized over social cues for enhancing knowledge retention.

## 1 Introduction

As digital transformation in education advances, the use of digital educational resources, particularly instructional videos, has increased. However, their practical application outcomes remain less than ideal (Shek et al., 2023). In recent years, a vast amount of online information and short-form video social media has permeated students’ lives. This fragmented, highly stimulating information environment gradually impairs students’ sustained attention and selective attention, which are essential for engaging with instructional videos that require focused mental processing (Chen et al., 2023). Drawing students’ attention has been a key instructional design concern in learning research.

To counter this, cues play an essential role in guiding students’ attention in learning. Researchers have suggested two main cue types: visual and social cues (Moon and Ryu, 2021). Incorporating visual and social cues into instructional videos can effectively focus students’ attention and enhance their learning performance (King et al., 2023; Wang et al., 2020). Nevertheless, the conditions under which these two types of cues foster learning remain unclear (Moon and Ryu, 2021). Moreover, individual differences may influence the effectiveness of visual and social cues (Arslan-Ari et al., 2020). While previous studies have focused more on students’ prior knowledge (Arslan-Ari, 2018), systematic research on individual differences in selective and sustained attention remains limited. The present study therefore investigates how visual versus social cues interact with learners’ sustained or selective attention levels to shape learning performance.

Instructional videos rank among the most widely used digital learning resources, as they present teaching materials in both visual and auditory formats (Pi et al., 2017). However, when students engage in learning through instructional videos, they often encounter difficulties in sustaining attention and exerting selective attention. To understand the nature of these difficulties, it is necessary to examine the construct of attention itself.

Attention is the mental process through which individuals selectively register some stimuli and ignore others (Boersma and Das, 2008). Previous research has explored how various forms of attention influence learning outcomes (Kokoç et al., 2020). Evidently, attention is of vital importance for students’ learning. Students’ attention levels may represent a significant internal factor that limits their outcomes in video-learning environments. Many students struggle to sustain their attention effectively during learning processes. Sustained and selective attention are, in particular, related to learning (Posner and Rothbart, 2007), and this study focused on these two attention types. Existing research has extensively examined the differences in learning outcomes between students differing in their levels of sustained or selective attention (Desjarlais, 2013; King et al., 2023; Kokoç et al., 2020).

Specifically, sustained attention is defined as the ability to continuously direct focus on a single task over a certain period (Roda, 2011). Sustained attention develops rapidly between the ages of 10 and 16 years and gradually becomes stable during early adulthood (Fortenbaugh et al., 2015). This type of attention is a key part of the broader attention system and is essential for effective learning processes (Adler and Orprecio, 2006). Studies in learning contexts reveal that sustained attention directly contributes to academic achievement (Steinmayr et al., 2010). In instructional video learning, high sustained attention demonstrates clear advantages over low sustained attention in information selection, sustained focus on the main content, efficiency of video processing strategies, and resulted in fewer unsuccessful actions (Desjarlais, 2013). Thus, enhancing instructional video learning for students with low sustained attention requires further consideration.

In contrast to sustained attention, selective attention is defined as the ability to focus on relevant stimuli while disregarding distractors or irrelevant information (Páez-Maldonado et al., 2020). Selective attention matures and stabilizes in early adulthood (Veer et al., 2017). Focusing attention on key information within a limited timeframe is critical when students learn from instructional video (Ilgaz et al., 2014). As a result, individuals possessing higher levels of selective attention can better focus on lesson-relevant information, whereas those with lower levels are more vulnerable to poor academic performance (Hanley et al., 2017; Rey, 2014). Thus, enhancing instructional video learning for students with low selective attention also requires additional research.

In summary, the optimization of instructional video learning for students with low sustained or selective attention requires further investigation. Previous studies have examined instructional aids, such as cues, that support student learning by guiding visual attention in multimedia instructional videos (Kestin and Miller, 2022).

Cueing refers to the use of signals that guide learners’ attention to essential information during instruction, ensuring that critical content is prioritized while distractions are minimized (Mayer, 2009). The attention-guiding principle (Jamet, 2014; Ozcelik et al., 2010) offers a straightforward explanation for cueing effects: cues added to learning materials direct attention to specific elements, thereby enhancing their processing (Scheiter and Eitel, 2015). Thus, the addition of cues can be regarded as an effective instructional strategy as it minimizes learners’ time spent on information search, thereby freeing working memory resources for learning (Mayer, 2009; Scheiter and Eitel, 2015). These cues can be broadly classified into two main types: visual and social cues (Moon and Ryu, 2021). Visual cues are intended to present visual information and highlight task information, for example by using color-coding. Visual cues direct the learners’ gaze to deliberately move to locations where key visual stimuli are presented in multimedia instruction (Moon and Ryu, 2021). Social cues, on the other hand, seek to enhance learners’ attention by fostering social interaction with the instructor, including gestures, eye contact and body orientation (Moreno et al., 2010).

Theoretically, the effectiveness of visual and social cues can be explained from two perspectives: the cognitive theory of multimedia learning (CTML; Mayer, 2009) and the social agency theory (SAT; Mayer and DaPra, 2012). The CTML holds that visual cues assist learners in quickly picking out key information, cutting down the search for irrelevant content, reducing cognitive load, and facilitating in-depth processing of learning materials (Brouwers et al., 2016). Instructional videos deliver abundant dynamic graphics, text, audio, and visual content, requiring learners to sift through massive information within a limited time and fix their attention on core knowledge (Ilgaz et al., 2014). Hence, visual cues highlighting key content can effectively steer learners’ attention toward vital information, streamline information screening and lessen redundant cognitive burden. Existing studies have proven that students with low selective attention are highly susceptible to distracting stimuli (Hanley et al., 2017) and students with high selective attention perform better on a skill achievement test (King et al., 2023). We speculate that visual cues marking key information can guide learners with low selective attention to relevant details, reduce their cognitive load and boost learning performance. In contrast, students with high selective attention possess stronger inherent filtering capacity and rely far less on such cue in instructional videos.

The second perspective, SAT, suggests that social cues boost learning performance by activating students’ social responses. In the context of instructional video design, enhancing social presence is a crucial aspect of optimizing learning outcomes (Kreijns et al., 2022). According to this theory, social cues promote students’ social attention via social conversation schemas. In environments enhanced with social cues, students respond more actively to instructors and engage in social processing, which enhances their social presence (Khan et al., 2021). Kokoç et al. (2020) revealed that students with high sustained attention achieved better learning outcomes than those with low sustained attention when learning from the same instructional videos. Their eye movement data also showed that students with low sustained attention made more fixations and had longer fixation durations on the instructor’s area of interest. Accordingly, we speculate that learners with low sustained attention pay more attention to the instructor during the process of acquiring new knowledge. Social cues such as eye contact and gestures allow them to develop parasocial interaction with the instructor. This interaction enhances social presence and promotes learning. In contrast, students with high sustained attention are less likely to be affected by the cues presented in instructional videos.

Prior research has consistently validated the effectiveness of embedding visual or social cues in learning materials such as instructional videos relative to cue-free conditions (De Koning et al., 2007; Mautone and Mayer, 2001). Arslan-Ari (2018) demonstrated that low-prior-knowledge students benefited from visual cues, outperforming their peers who learned without such cues. Ozcelik et al. (2009) reported that learners exposed to visually cued materials (i.e., color-coded formats) outperformed those who studied the same content in an uncued (conventional) format on both retention and transfer measures. Additionally, incorporating social cues into instructional videos enhances learning outcomes. Meier et al. (2023) observed that participants processed relevant information faster and achieved better learning outcomes when relying on social cues in low visual complexity conditions compared with high visual complexity conditions. Pi et al. (2017) reported that a teacher’s pointing gestures—as a form of social cue—enhanced learning performance to a greater extent than nonhuman cues or the absence of cues.

However, only one study explored the effectiveness of visual and social cues (Moon and Ryu, 2021). Notably, the social cues in this study were limited to the beat gestures of the cartoon pedagogical agent. Further exploration is needed to determine whether one type of cue is superior to the other. Moreover, individual differences may influence the effectiveness of visual and social cues (Arslan-Ari et al., 2020). Previous studies have focused more on students’ prior knowledge (Arslan-Ari, 2018), with insufficient consideration of the potential individual differences arising from variations in students’ sustained and selective attention levels. This gap in current research warrants further investigation. Only by identifying the types of cues suited to different attention characteristics can students be provided with precise video optimization solutions.

Drawing on the foregoing analyses, the following research questions were posed (RQs):

*RQ1*: How do visual and social cues added to instructional videos differentially impact the learning outcomes of students with differing levels of sustained attention?

*RQ2*: How do visual cues and social cues added to instructional videos differentially impact the learning outcomes of students with varying levels of selective attention?

To address RQ1 and RQ2, two experiments were conducted. Specifically, selective and sustained attention represent relatively independent dimensions of attention, which gradually reach relative maturity in early adulthood, namely the university years (Luna et al., 2004). Prior research confirms that notable, consistent individual differences in attention remain prominent and stable even once these capacities are fully mature (King et al., 2023; Unsworth and Miller, 2021). Hence, further research into this area is justified after both forms of attention mature. Consequently, as university students are in early adulthood, we recruited undergraduates as participants in this experiment. Moreover, the “global versus local processing” paradigm, proposed in Navon’s (1977) seminal study on perceptual processing, was chosen as representative instructional content. This topic was chosen because it is typically an unfamiliar, appropriately difficult cognitive psychology topic that can be delivered as a structured instructional video, analogous to a real-world online video lesson. Based on CTML and SAT, this study examined not only students’ test scores but also their perceived cognitive load and social presence levels.

Experiment 1 targeted students with varying levels of sustained attention. It aimed to determine which cue is most beneficial for students with low sustained attention. This experiment compared visual and social cue effects on learning outcomes, cognitive load, and social presence in instructional videos, examining differences between students with high and low sustained attention. Guided by SAT, we hypothesized that social cues increase social presence among students with low sustained attention. This enhancement, in turn, optimizes their learning performance. Therefore, the following hypotheses were proposed:

*H1:* For students with low sustained attention, social cues versus visual cues significantly enhance social presence, retention, and transfer test scores, while no significant difference is expected in cognitive load.

*H2:* For students with high sustained attention, social and visual cues show no significant differences in their effects on retention and transfer test scores, social presence, or cognitive load.

Experiment 2 targeted students with varying levels of selective attention. It aimed to determine which cue is most beneficial for students with low selective attention. This experiment compared visual and social cue effects on learning outcomes, social presence, and cognitive load in instructional videos, examining differences between students with high and low selective attention. Guided by CTML, we hypothesized that visual cues effectively reduce the cognitive load of students with low selective attention, thereby optimizing their learning performance. Therefore, the following hypotheses were proposed:

*H3*: For students with low selective attention, visual cues versus social cues significantly reduce cognitive load, enhance retention and transfer test scores, while no significant difference is expected in social presence.

*H4*: For students with high selective attention, visual and social cues show no significant differences in their effects on retention and transfer test scores, social presence, or cognitive load.

## 2 Experiment 1

### 2.1 Methods

#### 2.1.1 Participants

A sample of 210 participants was recruited from a university in China. Thirteen participants who did not pass the pretest were excluded. Subsequently, 197 participants were assigned to complete the sustained attention test. The top and bottom 27% of participants (based on sustained attention test scores) were selected for the formal experiment (Preacher, 2015), resulting in a final sample of 105 participants. The participants ranged from 17 to 21 years old (52 men, 53 women; mean age = 18.46 years, *SD* = 0.62). All participants voluntarily participated in the experiment, and each participant received a small gift as an incentive after the experiment concluded. This study was approved by the Research Ethics Committee of Ludong University (no. LDU-IRB202406014), with written informed consent obtained from all participants.

#### 2.1.2 Design

A 2 (sustained attention level: high, low) × 2 (cue type: visual, social) between-participants design was adopted. The dependent variables included the following: (1) learning performance: retention test score and transfer test score; (2) subjective scales: cognitive load and social presence.

#### 2.1.3 Materials

##### 2.1.3.1 Pretest materials

First, participants were asked whether they had been previously exposed to the to-be-learned content. Then, they were required to complete a pretest regarding the to-be-learned material, which consisted of two parts: retention and transfer. The retention section included 12 fill-in-the-blank items worth 1 point each, for a total of 12 points. The transfer section comprised eight fill-in-the-blank questions (1 point each) and one short-answer question (2 points), yielding 10 points. An earlier pilot pretest was conducted to establish the cutoff scores in the formal pretest. Results showed that the scores of participants who had never learned the experimental content ranged from 0 to 5 (out of 22), with a mode of 0. Accordingly, 5 points were set as the cutoff score in the formal pretest to select eligible participants who were novices in the to-be-learned material.

##### 2.1.3.2 Sustained attention test materials

**FIGURE 1:**
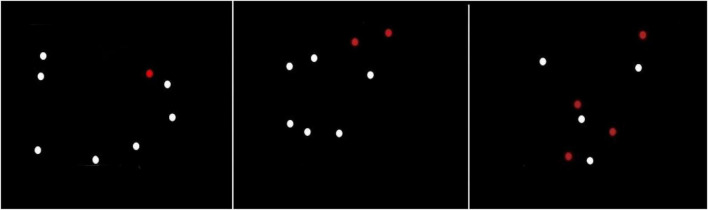
Sample images of the sustained attention test (from left to right as follows: easy, medium, and difficult).

To measure participants’ sustained attention levels, we employed a computerized assessment that was developed in accordance with the Yale University multiple object tracking paradigm (Kokoç et al., 2020). It has been extensively applied to measure sustained attention in e-learning research, as it requires attention to be maintained over a period of time (Ilgaz et al., 2014). In our experiment, the test was conducted using E-Prime 3.0 software. The task consisted of the following steps: participants completed one practice session, after which they undertook three ball-tracking sessions graded by difficulty (easy, medium, and difficult). A practice session was first conducted to familiarize participants with the task. Eight balls appeared on the main screen after practice session. At each difficulty level, a specific number of balls flashed to indicate the targets participants needed to track. All balls then moved randomly across the screen for 1 s. The sustained attention test required participants to track moving balls and identify designated targets once the balls came to a stop. Three difficulty levels were administered: easy (one target among eight balls), medium (two targets), and difficult (four targets). Each level was repeated 10 times, with scores ranging from 0 to 10 per level. 4–5 min per stage resulted in a total test duration of roughly 20 min. Sample screenshots are provided in Figure 1.

##### 2.1.3.3 Learning materials

**FIGURE 2:**
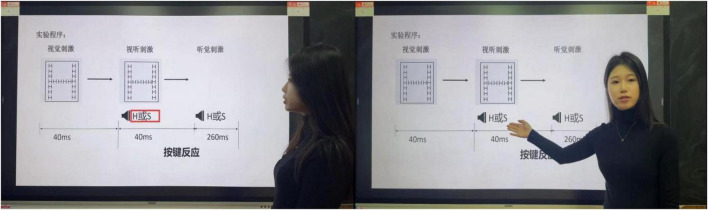
Screenshot of instructional video adding visual cues (left) and social cues (right).

The instructional video focused on the “global versus local processing” paradigm as its central instructional content. A real person recorded the instructional video in a classroom to explain the relevant learning content. This study operationalized the experimental manipulations for visual and social cues in a learning context. This operationalization follows established procedures in the literature (Meier et al., 2023; Moon and Ryu, 2021). Specifically, in instructional videos employing visual cues, these visual cues were implemented as red borders highlighting the area near either the text or pictorial content of the learning material (Moon and Ryu, 2021). The instructor faced sideways throughout the session; in this manner, any hand gestures were prohibited and interaction with students was avoided, thereby ensuring that social cues such as pointing gestures and eye contact were excluded. In instructional videos employing social cues, social cues were designed as gestures and eye contact that emphasized the moments associated with key information in the narration (Meier et al., 2023). The instructor faced the students directly throughout the session (see Figure 2). To ensure experimental rigor, the instructor’s verbal expressions and content maintained a high degree of consistency across the two experimental conditions. Moreover, the 29 visual and social cues were strictly matched in both frequency and presentation duration between the two conditions. The camera was positioned approximately 135 cm from the instructor, consistent with the interpersonal distance suggested by Sorokowska et al. (2017). Both instructional videos lasted 7 min and 25 s, and were displayed on a 22-inch monitor. Written consent for teacher portrait use in experiments obtained.

##### 2.1.3.4 Posttest materials

Upon viewing the instructional video, participants underwent a series of tests to assess their learning outcomes and completed subjective scales. Details are as follows:

###### 2.1.3.4.1 Retention and transfer tests

Referring to previous studies (Ploetzner and Schlag, 2013), the retention and transfer posttests were the same as the pretest. The retention test comprised 12 fill-in-the-blank questions (1 point each, scores ranging from 0 to 12 points, with higher scores indicating better retention), while the transfer test consisted of eight fill-in-the-blank questions (1 point each) and one short-answer question (2 points, yielding a total score ranging from 0 to 10 points), for a total of 10 points. Points were assigned for correct answers; incorrect or unanswered questions did not result in point deductions. Two independent raters with high inter-rater reliabilities (*r* = 0.99 and 0.98, respectively; *p*s < 0.001) evaluated the answers. Given the extremely high consistency of the two sets of scores, the complete scoring data was adopted from one trained rater for final statistical analysis and data processing while ensuring scoring validity and reliability.

###### 2.1.3.4.2 Cognitive load scale

The Cognitive Load Scale developed by Paas (1992) was used (Cronbach’s α = 0.74). This questionnaire has been widely used in the field of cognitive load measurement. It contains two 7-point scale questions, which measure the difficulty of learning materials as perceived by the learners (1 = very easy, 7 = very difficult) and the degree of mental effort expended in learning as perceived by the learners (1 = least effort, 7 = maximum effort). This scale, which has been widely used to measure students’ cognitive load in instructional video lectures, was employed to calculate cognitive load scores based on the summed ratings of its two items (Hong et al., 2018; Sweller et al., 2019).

###### 2.1.3.4.3 Social presence scale

The Social Presence Scale revised by Short et al. (1976) was used (Cronbach’s α = 0.65, 7-point scale). It consists of eight questions, with Questions 4, 6, and 8 being reverse scored. Higher scores indicate that university students have a higher sense of social presence during their learning.

### 2.2 Procedure

**FIGURE 3:**
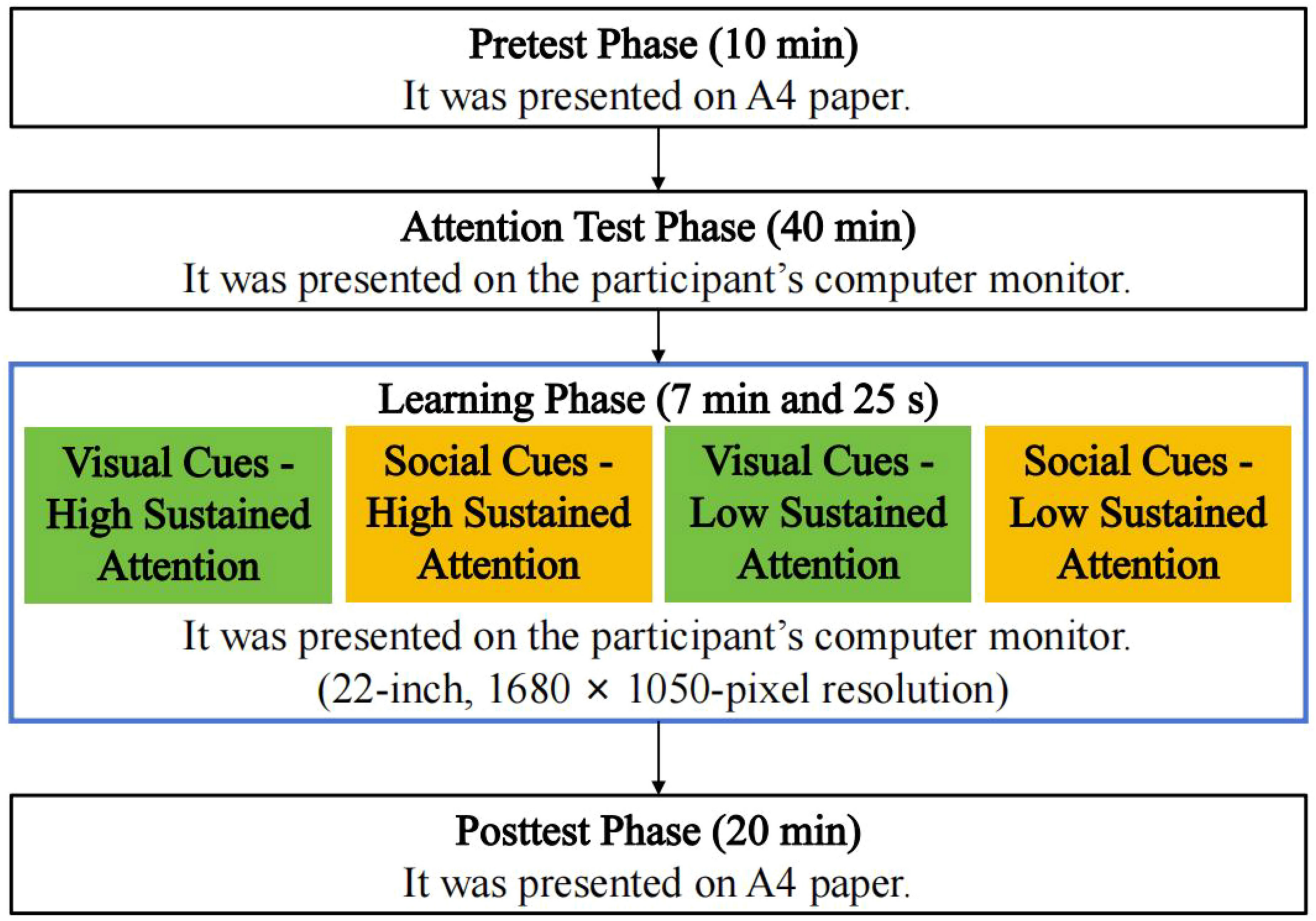
Experimental procedure flowchart.

Participants worked independently on their own materials throughout the procedure under the instruction and supervision of experimental assistants. No feedback was provided. The general procedure is illustrated in Figure 3.

The pretest phase lasted 10 min. In the attention test phase, the participants completed a multi-target tracking task lasting approximately 20 min. The participants were grouped into high and low sustained attention groups (top 27% and bottom 27%) based on the accuracy rates across the three conditions (easy, moderate, and difficult) and the calculated average accuracy rate. To minimize extraneous variables effectively, the participants were asked to complete a selective attention test to ensure that they all demonstrated moderate levels of selective attention (lasting approximately 20 min, see Experiment 2 for details of the test). The learning phase, in which experimental variations occurred, followed. Participants with high sustained attention and low sustained attention were randomly assigned to either the social cue or visual cue conditions, respectively. Consequently, four groups were constructed: visual cues-high sustained attention; social cues-high sustained attention; visual cues-low sustained attention; and social cues-low sustained attention, with each group watching its respective instructional video. Subsequently, participants were allowed up to 20 min to complete the posttests.

### 2.3 Results

To assess the validity of the grouping, we conducted an independent samples *t*-test to compare sustained attention scores between the high and low groups. The results indicated that students with high and low sustained attention exhibited significant differences in sustained attention scores, [*t*(103) = 15.60, *p* < 0.001, *d* = 3.04, 95% CI (2.48, 3.61)].

There was no significant difference in selective attention scores between the high sustained attention group and the low sustained attention group, [*t*(103) = 0.31, *p* = 0.761, *d* = 0.06, 95% CI (−0.32, 0.44)]. No significant age differences were found among the four groups of participants, [*F*(3, 101) = 1.54, *p* = 0.209, *η_*p*_*^2^ = 0.04, 95% CI (0.00, 0.12)]. Additionally, no significant differences in gender distribution, [χ^2^(3) = 2.42, *p* = 0.490, Cramér’s V = 0.15, 95% CI (0.07, 0.38)], or in pretest scores, [*F*(3, 101) = 0.05, *p* = 0.987, *η_*p*_*^2^ = 0.001, 95% CI (0.00, 0.006)], were found. Thus, the four groups were comparable in terms of age, gender, and pretest scores.

A two-factor analysis of variance was conducted with cue type and sustained attention level as independent variables, and participants’ retention and transfer test scores, cognitive load, and social presence as dependent variables. Table 1 shows descriptive statistics.

**TABLE 1:** Mean performance of the test, the ratings of cognitive load and social presence in Experiment 1.

Sustained attention level	Cue type	*n*	Retention test scores		Transfer test scores		Cognitive load		Social presence	
			*M*	*SD*	*M*	*SD*	*M*	*SD*	*M*	*SD*
High sustained attention	Visual	28	5.39	3.35	3.21	2.23	8.75	1.97	31.43	4.91
	Social	24	5.46	2.77	3.00	2.47	8.67	1.71	29.29	6.43
Low sustained attention	Visual	24	6.04	2.39	2.42	2.04	8.42	2.17	31.71	5.81
	Social	29	6.59	2.57	4.21	2.14	9.03	2.40	31.03	5.90

For the retention test scores, the main effect of sustained attention level was not significant, [*F*(1, 101) = 2.61, *p* = 0.109, *η_*p*_*^2^ = 0.03, 95% CI (0.00, 0.08)], and the main effect of cue type was also not significant, [*F*(1, 101) = 0.31, *p* = 0.580, *η_*p*_*^2^ = 0.003, 95% CI ([0.00, 0.04)]. The interaction between sustained attention level and cue type was not significant, [*F*(1, 101) = 0.19, *p* = 0.664, *η_*p*_*^2^ = 0.002, 95% CI (0.00, 0.04)].

For transfer test scores, the main effect of sustained attention level was not significant, [*F*(1, 101) = 0.22, *p* = 0.640, *η_*p*_*^2^ = 0.002, 95% CI (0.00, 0.04)], and the main effect of cue type was also not significant, [*F*(1, 101) = 3.27, *p* = 0.073, *η_*p*_*^2^ = 0.03, 95% CI (0.00, 0.10)]. However, the interaction between sustained attention level and cue type was significant, [*F*(1, 101) = 5.29, *p* = 0.023, *η_*p*_*^2^ = 0.05, 95% CI (0.01, 0.14)]. Simple effects analysis determined that for students with low sustained attention, transfer test scores in the social cue group were significantly higher than those in the visual cue group, [*F*(1, 101) = 8.51, *p* = 0.004, *η_*p*_*^2^ = 0.08, 95% CI (0.02, 0.18)]. By contrast, for students with high sustained attention, no significant difference was found in the transfer scores between the two cue groups, [*F*(1, 101) = 0.12, *p* = 0.730, *η_*p*_*^2^ = 0.001, 95% CI (0.00, 0.02)].

For the cognitive load, the main effect of sustained attention level was not significant, [*F*(1, 101) = 0.002, *p* = 0.967, *η_*p*_*^2^ < 0.001, 95% CI (0.00, 0.001)]. The main effect of cue type was also not significant, [*F*(1, 101) = 0.43, *p* = 0.516, *η_*p*_*^2^ = 0.004, 95% CI (0.00, 0.05)]. The interaction between sustained attention level and cue type was not significant, [*F*(1, 101) = 0.73, *p* = 0.394, *η_*p*_*^2^ = 0.01, 95% CI (0.00, 0.06)].

For social presence, the main effect of sustained attention level was not significant, [*F*(1, 101) = 0.80, *p* = 0.372, *η_*p*_*^2^ = 0.01, 95% CI (0.00, 0.07)]. The main effect of cue type was also not significant, [*F*(1, 101) = 1.55, *p* = 0.216, *η_*p*_*^2^ = 0.02, 95% CI (0.00, 0.09)]. The interaction between sustained attention level and cue type was not significant, [*F*(1, 101) = 0.42, *p* = 0.518, *η_*p*_*^2^ = 0.004, 95% CI (0.00, 0.05)].

### 2.4 Summary of experiment 1

As shown by the results of Experiment 1, transfer test scores can be improved when social cues rather than the visual cues are presented in instructional videos. The experimental findings partially supported H1, with validation observed only in the transfer test scores. The retention test score analysis failed to support the hypothesis, and neither cognitive load nor social presence showed significant differences. However, for students with high sustained attention, no difference was found between social and visual cue effects, which was consistent with H2. Based on this, Experiment 2 further examined the effectiveness of visual and social cues for students with varying levels of selective attention.

## 3 Experiment 2

### 3.1 Methods

#### 3.1.1 Participants

A sample of 198 participants was recruited from a university in China. Six participants who did not pass the pretest were excluded. Subsequently, 192 participants were assigned to complete the selective attention test. The top and bottom 27% of participants (based on selective attention test scores) were selected for the formal experiment, resulting in a final sample of 104 participants. The participants ranged from 17 to 20 years old (46 men, 58 women; mean age = 18.44 years, *SD* = 0.64). All participants voluntarily participated in the experiment, and each participant received a small gift as an incentive after the experiment concluded. This study was approved by the Research Ethics Committee of Ludong University (no. LDU-IRB202406014), with written informed consent obtained from all participants.

#### 3.1.2 Design

A 2 (selective attention level: high, low) × 2 (cue type: visual, social) between-participants design was adopted. The dependent variables included the following: (1) learning performance: retention test score and transfer test score; (2) subjective scales: cognitive load and social presence.

#### 3.1.3 Materials

The pretest materials, learning materials, and posttest materials were identical to those adopted in Experiment 1. Two independent raters with high inter-rater reliabilities (*r* = 0.99 and 0.99, respectively; *p*s < 0.001) evaluated the answers.

**FIGURE 4:**
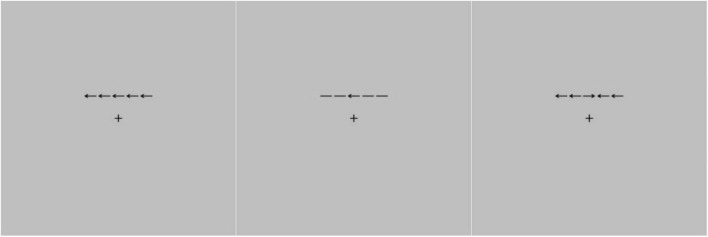
Illustration of the three trial types presented during the selective attention test (from left to right as follows: congruent conditions, neutral conditions, and incongruent conditions).

To measure participants’ selective attention levels, a computer-based test developed according to the Flanker task paradigm was used. Though the Flanker task primarily measures inhibitory and executive control, this inhibitory filtering function is also a core subcomponent of selective attention. King et al. (2023) and McDermott et al. (2007) employed the Flanker paradigm as a standard behavioral assessment of individual selective attention differences in cognitive and educational research. Selective attention fundamentally requires suppressing task-irrelevant distractors; the conflict inhibition quantified by the Flanker task directly reflects this core mechanism. The experiment was conducted using E-Prime 3.0 software. The stimuli consisted of a central target arrow flanked by two distractor arrows on each side. The distractors either pointed in the same direction as the central arrow (congruent condition), the opposite direction (incongruent condition), or appeared as horizontal lines without directionality (neutral condition), as illustrated in Figure 4. This formed a single row of five arrows, occupying 0.05% of the display. Target and distractor arrows were presented 1 cm above or below a central fixation cross. All stimuli were black against a gray background. Each trial began with a central fixation displayed for a variable duration (1,000, 1,500, 2,000, or 2,500 ms), followed by the arrows appearing above or below the fixation for 350 ms. Participants had 1,700 ms from stimulus onset to respond. They were instructed to maintain focus on the central fixation and indicate the direction of the central arrow using key presses (“J” for left, “K” for right). Across conditions, equal numbers of trials featured arrows above versus below the fixation, and the central arrow pointed left versus right with equal frequency. The test lasted approximately 20 min.

### 3.2 Procedure

The participants worked independently on their own materials throughout the procedure under the instruction and supervision of experimental assistants. No feedback was provided. The procedures were essentially identical to those in Experiment 1.

The pretest phase lasted 10 min. In the attention test phase, the participants completed a Flanker task lasting approximately 20 min. Based on their accuracy rates across the three conditions (congruent, neutral, and incongruent) and the calculated average accuracy rate, the participants were grouped into high and low selective attention groups according to the top 27% and bottom 27% of the scores. To validate the effectiveness of selective attention grouping, the participants completed a sustained attention test to ensure that all selected participants demonstrated moderate levels of sustained attention (see Experiment 1 for details of the test). The learning phase, in which experimental variations occurred, followed. Participants with high selective attention and low selective attention were randomly assigned to either the social cue or visual cue conditions, respectively. Consequently, four groups were constructed: visual cues-high selective attention; social cues-high selective attention; visual cues-low selective attention; and social cues-low selective attention, with each group watching its respective instructional video. Subsequently, participants were allowed up to 20 min to complete the posttests.

### 3.3 Results

To assess the validity of the grouping, we conducted an independent samples *t*-test to compare selective attention scores between the high and low groups. The results indicated that students in the high and low selective attention groups exhibited significant differences in their selective attention scores, [*t*(102) = 14.23, *p* < 0.001, *d* = 2.79, 95% CI (2.24, 3.33)]. No significant difference was found in the sustained attention scores between the two groups, [*t*(102) = 0.04, *p* = 0.971, *d* = 0.007, 95% CI (−0.38, 0.39)].

No significant differences in age were found between the four groups of participants, [*F*(3, 100) = 0.92, *p* = 0.436, *η_*p*_*^2^ = 0.03, 95% CI (0.00, 0.08)]. Additionally, no significant differences in gender distribution, [χ^2^(3) = 1.72, *p* = 0.634, Cramér’s *V* = 0.13, 95% CI (0.06, 0.35)], or in pretest scores, [*F*(3, 100) = 0.03, *p* = 0.993, *η_*p*_*^2^ = 0.001, 95% CI (0.00, 0.004)], were found.

A two-factor analysis of variance was conducted with cue type and selective attention level as independent variables, and participants’ retention and transfer test scores, cognitive load, and social presence as dependent variables. Table 2 shows descriptive statistics.

**TABLE 2:** Mean performance of the test, the ratings of cognitive load and social presence in Experiment 2.

Selective attention level	Cue type	*n*	Retention test scores		Transfer test scores		Cognitive load		Social presence	
			*M*	*SD*	*M*	*SD*	*M*	*SD*	*M*	*SD*
High selective attention	Visual	26	7.56	1.50	3.50	1.84	8.08	1.52	31.19	5.30
	Social	26	7.31	1.78	3.54	1.77	9.04	1.87	30.27	4.75
Low selective attention	Visual	26	7.38	2.08	2.92	2.23	8.62	1.98	32.19	3.60
	Social	26	5.51	2.57	3.46	2.14	8.65	2.04	29.65	7.53

For the retention test scores, the main effect of selective attention level was significant, [*F*(1, 100) = 9.33, *p* = 0.003, *η_*p*_*^2^= 0.09, 95% CI (0.03, 0.20)]. The main effect of cue type was also significant, [*F*(1, 100) = 10.55, *p* = 0.002, *η_*p*_*^2^ = 0.10, 95% CI (0.03, 0.22)]. The interaction between selective attention level and cue type was significant, [*F*(1, 100) = 5.65, *p* = 0.019, *η_*p*_*^2^= 0.05, 95% CI (0.01, 0.14)]. Simple effects analysis revealed that for students with low selective attention, retention test scores in the visual cue group were significantly higher than those in the social cue group, [*F*(1, 100) = 15.82, *p* < 0.001, η_p_^2^ = 0.14, 95% CI (0.05, 0.29)]. By contrast, for students with high selective attention, no significant difference in retention scores was found between the two cue groups, [*F*(1, 100) = 0.38, *p* = 0.539, *η_*p*_*^2^ = 0.004, 95% CI (0.00, 0.04)].

For transfer test scores, the main effect of selective attention level was not significant, [*F*(1, 100) = 0.69, *p* = 0.407, *η_*p*_*^2^ = 0.007, 95% CI (0.00, 0.06)]. The main effect of cue type was not significant, [*F*(1, 100) = 0.54, *p* = 0.465, *η_*p*_*^2^ = 0.005, 95% CI (0.00, 0.06)]. The interaction between selective attention level and cue type was not significant, [*F*(1, 100) = 0.41, *p* = 0.526, *η_*p*_*^2^ = 0.004, 95% CI (0.00, 0.05)].

For cognitive load, the main effect of selective attention level was not significant, [*F*(1, 100) = 0.04, *p* = 0.834, *η_*p*_*^2^ < 0.001, 95% CI (0.00, 0.001)]. The main effect of cue type was also not significant, [*F*(1, 100) = 1.87, *p* = 0.174, *η_*p*_*^2^ = 0.02, 95% CI (0.00, 0.10)]. The interaction between selective attention level and cue type was not significant, [*F*(1, 100) = 1.60, *p* = 0.209, *η_*p*_*^2^ = 0.02, 95% CI (0.00, 0.09)].

For social presence, the main effect of selective attention level was not significant, [*F*(1, 100) = 0.03, *p* = 0.858, *η_*p*_*^2^ < 0.001, 95% CI (0.00, 0.001)]. The main effect of cue type was also not significant, [*F*(1, 100) = 2.59, *p* = 0.111, *η_*p*_*^2^ = 0.03, 95% CI (0.00, 0.10)]. The interaction between selective attention level and cue type was not significant, [*F*(1, 100) = 0.56, *p* = 0.455, *η_*p*_*^2^ = 0.006, 95% CI (0.00, 0.06)].

### 3.4 Summary of experiment 2

In conclusion, these findings demonstrated that, in the retention test alone, students with low selective attention performed better with visual than with social cues. These findings partially supported H3. Specifically, this hypothesis was supported only for the retention test scores. The transfer test, cognitive load, and social presence scores failed to support this hypothesis. However, for students with high selective attention, social cues and visual cues shared roughly similar effects, which was consistent with H4.

## 4 Discussion

This study explored the effects of visual and social cues on learning using instructional videos among students with different attention levels. Several findings of this study are discussed.

Regarding RQ1, the findings provide direct evidence regarding the influence of visual and social cues on students with different levels of sustained attention. Experiment 1 found that compared with visual cues, social cues significantly improved the transfer test scores of university students with low sustained attention, which partly aligns with the SAT. The result of our experiment partially supports Davis’s (2018) conclusion that conversational gestures (social cues) facilitate students’ transfer and retention performance. However, in our experiment, the difference in retention test scores was not statistically significant. Learning performance may be enhanced by social cues through the activation of students’ social responses. Direct gaze and gestures may activate current social responses and deep cognitive processing strategies of students with low sustained attention, thereby further enhance transfer performance. Students’ motivation to learn increases when they feel that the instructor is approachable and supportive, thereby facilitating deeper understanding and knowledge transfer (Kuang et al., 2024). Moreover, social cues such as gestures might rely on the mirror neuron system to guide the trajectory of visual attention through the embodied simulation of instructors’ nonverbal behavior, thereby transforming abstract knowledge into operable cognitive units (Butera and Aziz-Zadeh, 2022) and facilitating deep knowledge integration and transfer. Therefore, we may speculate that for students with low sustained attention, although social cues facilitate comprehension during the encoding phase, they only improve students’ transfer performance, while failing to effectively enhance the retention of information into long-term memory. Experiment 1 found that visual and social cues showed no significant differences in primary learning outcome measures (transfer or retention scores) for university students with high sustained attention. One possible explanation is that high sustained attention may facilitate superior learning outcomes by enabling the effective allocation of attentional resources to the active selection of task-relevant information (King and Markant, 2020). Students with high sustained attention are more focused and perform better academically than those with low sustained attention (Kokoç et al., 2020); therefore, they are less influenced by external cues, which may account for the lack of significant statistical findings.

Regarding RQ2, the findings provide direct evidence regarding the influence of visual and social cues on students with different levels of selective attention. Experiment 2 found that compared with social cues, visual cues significantly improved the retention test scores of university students with low selective attention. This aligns in part with the CTML (Mayer, 2009). The potential reason might be as follows: Visual cues guide attention allocation directly through explicit visual prompts (Jamet, 2014; Ozcelik et al., 2009) and are therefore more beneficial for students with low selective attention. By helping students rapidly filter key information, visual cues reduce the attentional distraction caused by irrelevant stimuli. For students with low selective attention who may be limited in information processing capacity (Johnson et al., 2010), visual cues can simplify information processing, thereby enhancing memory retention. However, the advantages of visual cues did not extend to the transfer test. This is because their design primarily aids in the acquisition of foundational knowledge rather than guiding higher-order thinking (Xie et al., 2017). Although red-colored borders can assist in memorizing key terms, they may not prompt knowledge interconnectedness, making it difficult for students to transfer learned content to novel contexts (Hyönä and Lorch, 2004). Experiment 2 also found that visual and social cues showed no significant differences in primary learning outcome measures (transfer or retention scores) for university students with high selective attention. Therefore, we may speculate that visual cues are more conducive to memory retention among students with low selective attention. Students with high selective attention possess strong abilities in autonomous information filtering and concentration (King et al., 2023), which can offset the effects of cues. Therefore, they are less influenced by external cues, which explains why the statistical results were nonsignificant.

Cognitive load and social presence, which are important indicators of subjective learning processes, did not show significant main effects in either experiment. These finding contradicted our hypotheses. The finding of nonsignificant effects on cognitive load also contradicts the results reported by Rodemer et al. (2022), who demonstrated that incorporating cues into instructional videos effectively preserved participants’ cognitive resources, reduced extraneous cognitive load, and facilitated deeper information processing among students. The absence of a significant difference in cognitive load may be attributable to the inherent limitations of subjective measures. Factors such as the timing of assessments and recall bias can distort participants’ ratings, thereby diminishing the sensitivity of these measures to the actual cognitive load changes induced by the experimental procedures (Korbach et al., 2018). When self-reporting their cognitive load, participants may exhibit social desirability bias due to heightened concern about others’ perceptions of their performance; they may report higher levels of effort, even when they have not engaged diligently in the learning task (Zhang and Yang, 2022). In terms of social presence, the effect was nonsignificant. Several factors may explain this undesirable result. First, the depth and complexity of discussions influence the role of social presence (Gao et al., 2024). This study employed only the instructor’s gestures and gaze as social cues, without integrating multidimensional interactive elements such as vocal intonation and facial expressions, resulting in an insufficient perceived intensity of social presence. Second, the social presence rating employed in this study relies solely on participants’ subjective self-reports, which may show a lower sensitivity.

Notably, although certain observed performance differences are directionally consistent with CTML and SAT predictions, the lack of significant effects on the cognitive load and social presence means that these theoretical mechanisms were not empirically verified in the present study. Any causal inferences about how the cues work remain speculative. Future research should include more sensitive measures or larger samples to detect the theoretical mechanisms.

### 4.1 Limitations and future research directions

This study explored how visual and social cues influence learning outcomes among university students with different levels of attentional focus, revealing matching patterns between cue types and attentional characteristics. Furthermore, this research has several limitations that warrant further refinement and expansion in future studies.

Firstly, this study did not include a no-cue baseline condition, which limits our ability to determine absolute cue effectiveness. Additionally, our manipulation of social cues simultaneously varied multiple components (eye gaze, gestures, and body orientation), making it impossible to isolate the unique contribution of any single cue. Future research should include a no-cue control condition and employ factorial designs to disentangle the independent effects of gaze, gestures, and orientation.

Secondly, the measurements relied mainly on behavioral tests and subjective scales, which may lack sensitivity for variables such as cognitive load and social presence. Moreover, a relatively low level of reliability was reported for the social presence scales. Multimodal physiological data, such as EEG, eye-tracking, and electrodermal responses, should be incorporated and integrated into future research to provide more objective insights and verify that participants perceived or paid attention to the visual and social cues as intended.

Thirdly, the conclusions were drawn from a single university sample, short laboratory-based instructional videos, and specific topics, which limits their generalizability to real-world online learning. Longitudinal studies in naturalistic learning environments and across different participants (e.g., other populations or ages), learning tasks and instructional content are required to enhance ecological validity and generalization.

Finally, some results had small effect sizes. The application of findings to practice should be treated with due caution. Nonetheless, this remains a noteworthy effect to document because effects can cumulate over time, situations, or individuals, and thus, this small effect size might be potentially consequential in the long run.

## 5 Conclusion

For university students with low sustained attention, incorporating social cues into instructional videos was relatively more effective in promoting knowledge transfer than incorporating visual cues. For those with low selective attention, adding visual cues to instructional videos was relatively more effective in enhancing knowledge retention than adding social cues.

## Data Availability

The raw data supporting the conclusions of this article will be made available by the authors, without undue reservation.
